# Retro-orbital Metastasis in Chromophobe Renal Cell Carcinoma: A Case Report and Review

**DOI:** 10.34172/aim.31231

**Published:** 2025-03-01

**Authors:** Tanju Kapagan, Nilufer Bulut, Mehmet Halıcı, Gokmen Umut Erdem

**Affiliations:** ^1^Basaksehir Cam and Sakura City Hospital, Department of Medical Oncology, 34480 Istanbul, Turkey; ^2^Basaksehir Cam and Sakura City Hospital, Department of Radiation Oncology, 34480 Istanbul, Turkey

**Keywords:** Cancer, Chromophobe renal cell carcinoma, Palliative radiotherapy, Retro-orbital metastasis

## Abstract

Chromophobe renal cell carcinoma (ChRCC) is a rare subtype of renal cell carcinoma (RCC). We present a case of a 61-year-old male with ChRCC who exhibited unusual metastasis to the retro-orbital area, a site rarely affected by RCC. The patient experienced diplopia and proptosis, prompting treatment with sunitinib and palliative radiotherapy. Remarkably, rapid improvement in ocular symptoms was observed following radiotherapy. Despite this localized response, the patient’s overall condition deteriorated, highlighting the aggressive nature of ChRCC. This case underscores the importance of considering ChRCC in metastatic presentations and the potential efficacy of local palliative interventions.

## Introduction

 Chromophobe renal cell carcinoma (ChRCC) was first described in 1985 by Thoenes et al. ChRCC is a subcategory of renal cell carcinoma (RCC) that originates in the distal convoluted tubules.^1-3^ ChRCC affects females more often than males, and is most common during the fifth and sixth decades of life.^4^ ChRCC can mimic a broad spectrum of lesions, including oncocytoma and other subtypes of RCC.^5^ ChRCC can be distinguished from other RCC subtypes based on the cytoplasmic and nuclear changes observed during light microscopic examination, loss of specific chromosomes (as detected by high-resolution DNA-microarray analyses), and staining pattern of antibodies revealed by histological examination.^6^ ChRCC can be divided into three main subtypes: the eosinophilic variant (41%), the classical variant (12%), and the mixed variant (46%).^7^

 ChRCC rarely metastasizes. However, when it does metastasize, it often spreads to distant regions such as the liver, lungs, and skeletal system. Orbital metastasis is rare in RCC.^8,9^ Orbital metastases occur in 2%–5% of all cancer patients. Ocular or eye metastases usually arise from breast, lung, or prostate cancer.^10^ To the best of our knowledge, a ChRCC with orbital metastases has not been previously reported in the English-language literature.

 The current case is of interest not only for its rare metastatic features, but also its potential contribution to the palliative approach to the treatment of retro-orbital metastases.

## Case Report

**Figure 1:**
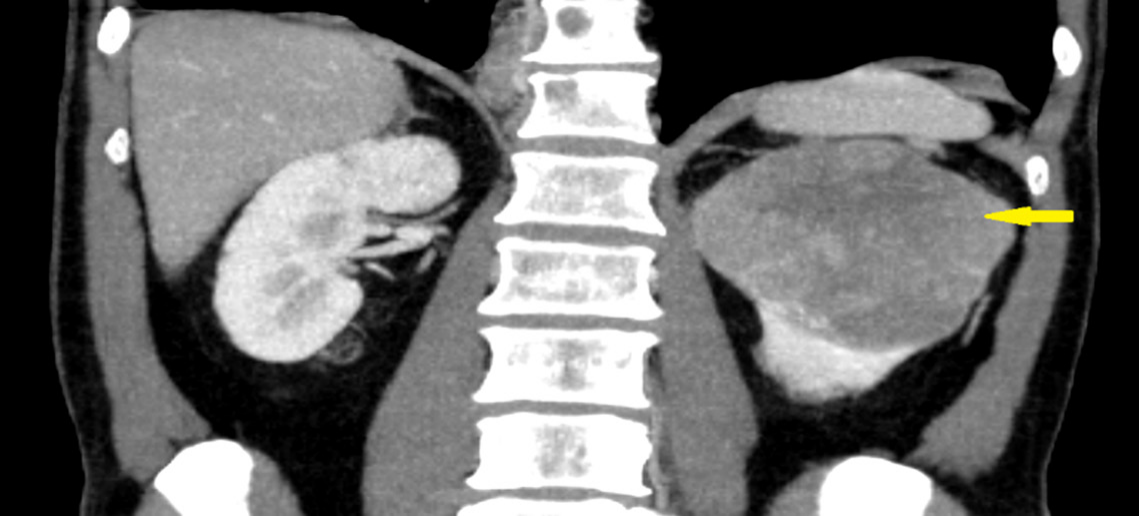
Renal Mass in Patient with Chromophobe Renal Cell Carcinoma

 A 61-year-old male was admitted to the internal medicine outpatient clinic with a 3-month history of left side pain. Upon physical examination, left costo-vertebral angle tenderness with diplopia and proptosis of the right eye were noted. All laboratory evaluations were normal except for the presence of microscopic hematuria. Urinary tract ultrasonography showed a solid lesion in the upper part of the left kidney with a diameter of 9 cm and areas of coarse heterogeneous calcification. Thoraco-abdominal computed tomography (CT) performed for metastatic workup revealed a primary 10 × 10.5 cm left kidney mass in the upper pole (Figure 1).

**Figure 2:**
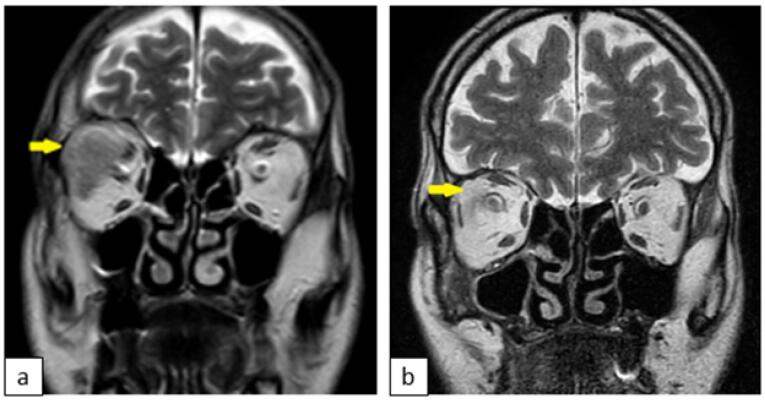
Retro-orbital Metastasis in Patient with Chromophobe Renal Cell Carcinoma **(a)** Before and **(b)** After Radiotherapy

 Multiple lytic destructive lesions were noted in the dorsal and lumbar vertebrae. Brain magnetic resonance imaging (MRI) showed a metastatic focus 20 × 15 mm in diameter in the right retro-orbital area (Figure 2a).

**Figure 3:**
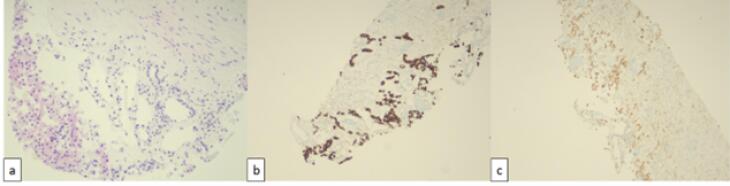
Histopathological Illustration of the Tumor Specimen Using H&E **(a)**, CK-7 **(b)** and PAX-8 **(c)**

 Renal mass biopsy of the lesion performed using a Tru-Cut 18-gauge needle under ultrasound guidance confirmed ChRCC (Figures 3a-3c).

 Based on these findings, we diagnosed stage IV disease ChRCC. The patient was referred for ophthalmology consultation because of ocular symptoms. A fundoscopic examination revealed limited lateral gaze in the right eye as a result of invasion of the rectus lateralis muscle in the retro-orbital region. Sunitinib was administered orally at a dose of 50 mg daily over 6-week cycles consisting of a 4-week treatment period followed by a 2-week rest period. In addition, the retro-orbital mass was targeted with palliative radiotherapy, i.e. external beam radiation therapy (EBRT) at a dose of 30 Gy. After radiation therapy, administration of systemic corticosteroids was initiated. This dramatically improved the proptosis, double vision, and ocular movements; all three symptoms disappeared within 3 months of completion of radiation therapy. MRI showed that the metastatic lesion in the right retro-orbital area had shrunk significantly (Figure 2b). However, control thoracoabdominal CT showed no significant difference in the renal mass or other metastatic foci. Given these results, the treatment protocol was changed to everolimus at 10 mg/d. The patient’s condition worsened, and he died 1 month later.

## Discussion

 According to the American Cancer Society, 76 080 and 3804 new cases of RCC and ChRCC were predicted to occur in the United States in 2021.^11^

 A study published in Annals of Surgical Oncology found that the rate of distant metastasis was 5.6% for ChRCC.^12^ However, after an extensive literature search, we found that the present case is the first report of retro-orbital metastasis of ChRCC. In a multicenter retrospective study, 10105 patients with confirmed diagnoses of RCC were evaluated in terms of histological subtype and distant metastasis. ChRCC is the RCC subtype most likely to metastasize, and can affect the liver, lungs, bones, adrenal gland, and peritoneum (in decreasing order of frequency). The liver is one of the most common sites for RCC metastasis, accounting for nearly 34% of all cases. In a multicenter retrospective study, 2% of patients had brain metastases. However, no retro-orbital metastases were identified in any patient.^13^ A retrospective study involving 124 patients with ChRCC and 1693 with clear-cell RCC was conducted at two centers from 1989 to 2006; 20 patients with ChRCC (16%) presented with metastases, but no brain or retro-orbital metastases were identified in any of the ChRCC patients.^14.^ ChRCC generally has a better prognosis and lower risk of metastasis than other RCC subtypes,^14-16^ but there was no significant difference between the groups in terms of metastasis.^14^

 In another retrospective study carried out at 10 centers, 36 patients with metastatic ChRCC were evaluated from 2004 to 2014; all patients received sunitinib as first-line treatment. The median progression-free survival was 10 months and the median overall survival was 26 months.^17^ The study was led by Dr. Andrew J. Armstrong from Duke University and the Duke Cancer Institute (Durham, NC, USA), and included a subgroup consisting of 16 patients with a diagnosis of metastatic ChRCC. Ten patients were treated with sunitinib and six with everolimus. Patients treated with everolimus had a higher partial/complete response rate compared with those treated with sunitinib (33% vs. 10%).^18^ In our patient, no significant improvement was observed in areas other than the metastatic focus in the retro-orbital area, where ERBT was applied as palliative treatment.

 Orbital metastases are an indicator of hematogenous spreading associated with a poor prognosis. Palliative care enhances the patient’s quality of life by controlling symptoms. In a meta-analysis conducted in Canada, orbital metastases were detected in 80 patients affected primarily by breast (29%), melanoma (20%), and prostate (13%) cancer. The most frequently reported symptoms were diplopia (48 %), followed by pain (42%) and vision loss (30%). The study found that radiotherapy can be very effective at relieving symptoms.^19^ Another retrospective study examined 15 patients with orbital metastases. After palliative radiotherapy using 20­–30 Gy doses in 10–15 fractions, all patients achieved a complete response in terms of eye pain and proptosis, and 84.6 % showed a partial response in terms of visual acuity.^20^

 Our patient received radiotherapy (30 Gy in 10 fractions). Three months after finishing radiation therapy, the patient was evaluated by an ophthalmologist who reported that the diplopia had regressed and the proptosis had disappeared.

## Conclusion

 Our case demonstrates that ChRCC, though rare, may behave aggressively and metastasize. It is important to be aware that the tumor can respond quickly to local palliative treatment. Further research is needed to characterize the sites and varieties of metastases; our case report provides a reference for such studies.
